# CRP-level-associated polymorphism rs1205 within the *CRP* gene is associated with 2-hour glucose level: The SAPPHIRe study

**DOI:** 10.1038/s41598-017-08696-2

**Published:** 2017-08-11

**Authors:** Wayne Huey-Herng Sheu, Wen-Chang Wang, Kwan-Dun Wu, Chih-Tsueng He, Chii-Min Hwu, Thomas Quertermous, Wan-Shan Hsieh, Wen-Jane Lee, Chih-Tai Ting, Yii-Der I. Chen, Chao A. Hsiung

**Affiliations:** 1 Division of Endocrinology and Metabolism, Department of Internal Medicine, Taichung Veterans General Hospital, Taichung, Taiwan; 2 College of Medicine, National Yang-Ming University, Taipei, Taiwan; 3 Institute of Medical Technology, National Chung-Hsing University, Taichung, Taiwan; 4 School of Medicine, National Defense Medical Center, Taipei, Taiwan; 5 The Ph.D. Program for Translational Medicine, College of Medical Science and Technology, Taipei Medical University, Taipei, Taiwan; 6 Division of Biostatistics and Bioinformatics, Institute of Population Health Sciences, National Health Research Institutes, Zhunan, Miaoli County, Taiwan; 7 Department of Internal Medicine, National Taiwan University Hospital, Taipei, Taiwan; 8 Division of Endocrinology & Metabolism, Tri-Service General Hospital, Taipei, Taiwan; 9 Section of Endocrinology and Metabolism, Department of Medicine, Taipei Veterans General Hospital, Taipei, Taiwan; 10 Faculty of Medicine, School of Medicine, National Yang-Ming University, Taipei, Taiwan; 11 Division of Cardiovascular Medicine, Falk Cardiovascular Research Building, Stanford University School of Medicine, Stanford, CA USA; 12 Department of Medical Research, Taichung Veterans General Hospital, Taichung, Taiwan; 13 Cardiovascular Center, Taichung Veterans General Hospital, Taichung, Taiwan; 14 Institute for Translational Genomics and Population Sciences and Department of Pediatrics, Los Angeles BioMedical Research Institute at Harbor-UCLA Medical Center, Torrance, California, USA

## Abstract

C-reactive protein (CRP) encoded by *CRP* gene is a reflection of systemic inflammation. Many studies associated CRP level with diabetes and glucose levels, but the association of *CRP* gene with these traits is unclear. We conducted a cross-sectional study consisting of 945 siblings from 330 families collected by the Stanford Asian Pacific Program in Hypertension and Insulin Resistance (SAPPHIRe) to investigate associations between *CRP* polymorphisms, circulating CRP, diabetes, and glucose levels. Five single-nucleotide polymorphisms were analyzed: rs3093059, rs2794521, rs1417938, rs1800947, and rs1205. The generalized estimating equation approach was used to deal with correlated data within families. CRP level was positively correlated with diabetes prevalence and levels of fasting and 2-hour glucose (each *P* < 0.008). Alleles *C* at rs3093059 and *G* at rs1205 were associated with elevated CRP level (each *P* < 1.2 × 10^−6^). Allele *C* at rs3093059 was associated with fasting glucose (*β* = 0.20, *P* = 0.045) and *G* at rs1205 was associated with 2-hour glucose (*β* = 0.46, *P* = 0.00090) post oral glucose tolerance test, but only the latter passed Bonferroni correction. No polymorphism was associated with diabetes. Since 2-hour glucose is an indicator of glucose tolerance, this study indicated *CRP* gene is associated with glucose intolerance.

## Introduction

Diabetes is a chronic, multifactorial disease characterized by hyperglycemia and has been one of the most important issues in public health worldwide^1^. Genetic factors play important roles in the pathogenesis of diabetes^2, 3^. Identification of the genetic determinants could facilitate the risk prediction of disease development and the implementation of individualized treatment for therapy. Up to now, genome-wide association studies (GWAS) for diabetes have identified more than 80 susceptibility loci, but only a small part of the heritability of diabetes can be explained by those findings^4^. On the other hand, as an alternative strategy to search for genetic factors of diabetes, recent genetic studies of diabetes-related quantitative traits have offered some new loci involved in the pathogenesis of diabetes^1, 5^. Nevertheless, more efforts have to be made to look for genetic variants accounting for the missing heritability of diabetes.

Accumulating evidence indicates that inflammation plays an important role in the pathogenesis of diabetes^6, 7^. Circulating C-reactive protein (CRP) concentration, an explicit reflection of systemic inflammation, has been associated with the risk of diabetes by ample studies in various cohorts^7^. Furthermore, CRP levels has also been shown to positively correlate with the levels of fasting and 2-hour glucose^8–10^, which are the major clinical measures in the diagnosis of diabetes^11^. Level of circulating CRP is highly heritable, with estimated heritability ranging from 22% to 56%^12–15^. Recent GWAS identified several genes associated with CRP levels; among those, the *CRP* gene had the strongest effect^16–19^. Several studies demonstrated the association between *CRP* polymorphisms and circulating CRP levels^20–23^.

Since circulating CRP is significantly associated with diabetes and glucose levels, we aimed to investigate whether *CRP* polymorphisms are associated with these traits. Up to now, however, the association of *CRP* gene with diabetes has been investigated by only a small number of studies and inconsistent results were reported^24–28^. On the other hand, a few studies investigated the association between *CRP* polymorphisms and glucose levels and almost all of these studies examined association with fasting but not postload glucose^29–32^. Furthermore, few studies investigating association of *CRP* polymorphisms with diabetes and glucose levels were conducted in population of Chinese ancestry.

The purpose of this study is to investigate associations between *CRP* single-nucleotide polymorphisms (SNPs), circulating CRP, diabetes, and levels of fasting and 2-hour glucose in a group of Taiwanese participants as part of the Stanford Asian Pacific Program in Hypertension and Insulin Resistance (SAPPHIRe). Firstly, we examined the associations between *CRP* SNPs and CRP level and between CRP level and diabetes as well as glucose levels. Then we investigated whether several SNPs of *CRP* gene are associated with diabetes and glucose levels.

## Results

The study sample consists of 945 siblings from 330 families collected in the SAPPHIRe follow-up study. The clinical characteristics of the study sample are summarized in Table 1. As described in Methods, six SNPs within the *CRP* gene were genotyped in the SAPPHIRe study. In the study population, 939 subjects have available genotype on at least one SNP and the proportion of successful genotyping of each SNP ranges from 98.3% to 99.2% (Table 2). By implementing the pairwise tagging option in Haploview software, with the default threshold of *r* ^2^ = 0.8, five of the six genotyped SNPs were selected as tags and were analyzed in this study: rs3093059, rs2794521, rs1417938, rs1800947, and rs1205.

**Table 1:** Clinical characteristics of the study sample.

Variable, unit	*N*	Mean ± SD/geometric mean [IQR]/percentage
Age, years	945	54.15 ± 8.89
BMI, kg/m^2^	943	25.69 ± 3.46
Waist, cm	943	89.38 ± 10.23
Fasting glucose, mmol/L	938	5.64 ± 1.75
2-hour glucose, mmol/L	840	8.79 ± 3.25
Systolic blood pressure, mm Hg	945	129.09 ± 22.27
Diastolic blood pressure, mm Hg	945	75.39 ± 11.65
Total cholesterol, mmol/L	935	5.19 ± 1.14
HDL, mmol/L	937	1.15 ± 0.35
Triglyceride, mmol/L	937	1.44 [0.97, 2.05]
CRP, mg/L	901	0.93 [0.49, 1.73]
Male, %	945	45.7%
Diabetes, %	914	28.4%
Medication for diabetes, %	945	8.8%
Hypertension, %	945	64.7%
Cardiovascular disease, %	945	11.6%
Current/ever smoking, %	945	27.9%
Current/ever alcohol drinking, %	945	28.6%
Physical activity, %	945	18.2%

*N*, number of subjects having available data; SD, standard deviation; IQR, interquartile range.

**Table 2:** *CRP* single-nucleotide polymorphisms (SNPs) genotyped in the SAPPHIRe study.

SNP	Chromosome position^a^	Gene region	MAF	*P* for HWE test	Number of subjects successfully genotyped
rs3093059	157951760	Promoter	0.17	0.75	937 (99.2%)
rs2794521	157951720	Promoter	0.19	0.11	933 (98.7%)
rs1417938	157950810	Intron 1	0.05	0.52	929 (98.3%)
rs1800947	157950062	Exon 2	0.08	0.32	934 (98.8%)
rs1205	157948857	3′ UTR	0.44	1.00	935 (98.9%)
rs3093077	157946260	3′ UTR	0.18	1.00	933 (98.7%)

MAF, minor allele frequency; HWE, Hardy-Weinberg equilibrium.

^a^Build 36.3.

To deal with the correlation between members within the same families, the generalized estimating equation (GEE) approach was used throughout the association analyses. Firstly, we examined the association of minor allele of individual tag-SNPs with the CRP level. We observed that rs3093059, rs1800947 and rs1205 are associated with CRP level but the p-value of rs1800947 does not pass the threshold required by Bonferroni correction (0.05  ÷  5 = 0.01) (Table 3). Specifically, increase of the log-transformed CRP level was significantly associated with the minor alleles *C* at rs3093059 (*β* = 0.31, *P* = 3.0 × 10^−7^) and *G* at rs1205 (*β* = 0.23, *P* = 1.2 × 10^−6^).

**Table 3:** Association of minor allele of *CRP* tag-SNPs with log-transformed CRP level.

SNP	Minor/Major allele	*β* (95%CI)^a^	*P*
rs3093059	*C*/*T*	0.31 (0.19, 0.42)	3.0 × 10^−7^
rs2794521	*G*/*A*	0.02 (−0.10, 0.13)	0.75
rs1417938	*T*/*A*	0.19 (−0.02, 0.39)	0.073
rs1800947	*C*/*G*	−0.18 (−0.34, −0.02)	0.031
rs1205	*G*/*A*	0.23 (0.14, 0.32)	1.2 × 10^−6^

^a^The association was adjusted for gender, age, BMI, waist, SBP, DBP, hypertension, diabetes, CVD, CHOL, HDL, TG, physical activity, smoking, and alcohol drinking.

Next, the association of circulating CRP level with the prevalence of diabetes, as well as the levels of fasting and 2-hour glucose, was investigated; the results are summarized in Table 4. We observed that the odds ratio (OR) of having diabetes associated with a unit increase of the log-transformed CRP level is 1.37 (*P* = 1.8 × 10^−4^). Furthermore, the increment of levels of fasting and 2-hour glucose associated with a unit increase of log-transformed CRP level are 0.13 mmol/L (*P* = 0.008) and 0.50 mmol/L (*P* = 9.6 × 10^−6^), respectively. It is obvious that the increment of 2-hour glucose level is larger than the increment of fasting glucose level and the association between circulating CRP and 2-hour glucose has the smallest p-value.

**Table 4:** Association of unit increase of the log-transformed CRP levels with diabetes prevalence and levels of fasting and 2-hour glucose.

Diabetes		Fasting glucose		2-hour glucose	
OR (95%CI)^a^	*P*	*β* (95%CI)^b^	*P*	*β* (95%CI)^b^	*P*
1.37 (1.16, 1.62)	1.8 × 10^−4^	0.13 (0.03, 0.23)	0.008	0.50 (0.28, 0.72)	9.6 × 10^−6^

^a^The association was adjusted for gender, age, BMI, waist, SBP, DBP, hypertension, CVD, CHOL, HDL, TG, physical activity, smoking, and alcohol drinking.

^b^The association was adjusted for gender, age, BMI, waist, SBP, DBP, hypertension, CVD, CHOL, HDL, TG, physical activity, smoking, alcohol drinking, and the use of medications for treating diabetes.

Since rs3093059 and rs1205 are associated with CRP level and CRP level is associated with diabetes prevalence and levels of fasting and 2-hour glucose, it is natural to investigate whether these two CRP-associated SNPs are associated with these traits. As shown in Table 5, minor allele *C* at rs3093059 is associated with elevated fasting glucose (*β* = 0.20, *P* = 0.045) and minor allele *G* at rs1205 is associated with elevated 2-hour glucose (*β* = 0.46, *P* = 0.00090). When applying the Bonferroni correction to deal with multiple testing, however, only the association between rs1205 and 2-hour glucose remained significant, according to the required threshold of p-value (0.05  ÷  6 ≈ 0.008). Both SNPs are not associated with diabetes prevalence.

**Table 5:** Association of minor allele of the CRP-associated SNPs with diabetes prevalence and levels of fasting and 2-hour glucose.

SNP (minor allele)	Diabetes		Fasting glucose		2-hour glucose	
	OR (95%CI)^a^	*P*	*β* (95%CI)^b^	*P*	*β* (95%CI)^b^	*P*
rs3093059 (*C*)	0.89 (0.63, 1.24)	0.48	0.20 (0.004, 0.39)	**0.045**	0.26 (−0.13, 0.64)	0.19
rs1205 (*G*)	1.08 (0.85, 1.37)	0.53	0.05 (−0.09, 0.19)	0.48	0.46 (0.19, 0.72)	**0.00090**

^a^The association was adjusted for gender, age, BMI, waist, SBP, DBP, hypertension, CVD, CHOL, HDL, TG, physical activity, smoking, and alcohol drinking.

^b^The association was adjusted for gender, age, BMI, waist, SBP, DBP, hypertension, CVD, CHOL, HDL, TG, physical activity, smoking, alcohol drinking, and the use of medications for treating diabetes.

We note that the observed relationships between rs1205 and levels of circulating CRP and 2-hour glucose are consistent in the sense that allele *G* at rs1205 is associated with elevated levels of CRP (Table 3) and 2-hour glucose (Table 5) and there is a positive correlation between these two quantitative variables (Table 4).

Furthermore, the family-based association test (FBAT) analysis, which has the advantage of avoiding confounding due to population stratification, was applied to significant SNP-trait associations observed in GEE analysis. In FBAT analysis, the p-values of one-sided test for the association of minor allele *G* at rs1205 with circulating CRP and 2-hour glucose levels are 0.020 and 0.0032, respectively (Supplementary Table S1). These results indicate that these significant associations observed in GEE analysis were not caused by population stratification.

## Discussion

Although many studies have reported a positive correlation between circulating CRP level and risk of diabetes^7^ as well as glucose levels^8–10^ and several *CRP* polymorphisms have been associated with CRP level^20–22^, up to now only a few studies have examined the association of *CRP* polymorphisms with diabetes and glucose levels^24–28^. In this study, we investigated the associations between *CRP* SNPs, circulating CRP, diabetes, as well as levels of fasting and 2-hour glucose based on a sample of 945 siblings collected in Taiwan, which is a population of Chinese ancestry. The observations that increased CRP level is significantly associated with higher diabetes prevalence and elevated glucose levels are in concordant with the results of previous studies^6–10^. The observed association of two SNPs, rs3093059 and rs1205, with circulating CRP is also reported by several previous studies^22, 33–37^.

Circulating CRP concentration is an explicit reflection of systemic inflammation. Circulating CRP not only indicates, through its level, the body’s responses to inflammatory or pathogenic stimuli but also actively participates in inflammation and atherosclerosis^38–40^. Circulating levels of CRP could be influenced by age, degree of obesity, sex, smoking status, and use of medications^41–43^. In addition to these environmental stimuli, it is well known that CRP concentration is also determined by genetic factors. The estimated heritability of CRP levels ranges from 22% to 56%^12–15^. Recent GWAS have identified several genes associated with CRP levels; among those, the *CRP* gene had the strongest effect^16–19^. Many genetic studies have demonstrated the association between *CRP* SNPs and circulating CRP levels^20–22^. In this study, we observed significant associations of rs3093059 and rs1205 with CRP levels (Table 3), which have also been reported previously^22, 33–37^.

Accumulating evidence indicates that inflammation plays an important role in the pathogenesis of diabetes^6, 7^. The association between circulating CRP level and diabetes has been reported by ample studies in various cohorts^7^. Consistent with the results of previous studies, this study showed that elevated CRP level is significantly associated with higher diabetes prevalence (Table 4). In addition to diabetes, CRP level has also been shown to be positively correlated with levels of fasting and 2-hour glucose^8–10^, which are major clinical measures in the diagnosis of diabetes^11^. We note that several studies have pointed out that circulating CRP is more strongly associated with 2-hour glucose than with fasting glucose^8, 9, 44^, and our study confirmed this relationship. Specifically, we observed that, for a unit increase of log-transformed CRP level, the increment of 2-hour glucose is about 3.8-fold higher than the increment of fasting glucose (0.50/0.13 ≈ 3.8, Table 4).

In contrast to circulating CRP level, the association of *CRP* polymorphisms with diabetes has been investigated by fewer studies and has remained controversial^24–28, 45^. Up to now, *CRP* has been associated with diabetes in populations from India^24, 46^, the Netherlands^25^, the United States^26^, and Greece^28^, but different associated polymorphisms were reported. For the five tag-SNPs analyzed in this study, Wolford *et al*. (2003) reported an association of rs2794521 in Pima Indians^24^ but it was not significant in several populations in the United States^26, 27^; Zee *et al*. (2008) showed an association of rs3093059 in a population of whites but not blacks in the United States^26^ and this association was not observed in another study in the United States^27^; an association of rs1205 was reported by Papaoikonomou *et al*. (2011) in Greece^28^ but it was not revealed in other populations^26, 27, 47^; an association of rs1800947 was examined by three studies^26, 27, 46^ but observed in an Indian population only^46^; no association of rs1417938 was reported previously. In the current study, no significant association of these five SNPs with diabetes was observed in a Taiwanese population. In addition to inconsistency between above association results, we would like to point out that almost all p-values of these associations were between 0.01 and 0.05^24, 26–28, 47^. The inconsistent and modest associations between *CRP* polymorphisms and diabetes observed in previous and current studies might be due to genetic heterogeneity of diabetes.

On the other hand, the association of *CRP* gene with glucose levels has been investigated by a small number of studies^29–32^. In this study, we observed that allele *C* at rs3093059 is associated with increased fasting glucose (*β* = 0.20, *P* = 0.045) and that allele *G* at rs1205 is associated with increased 2-hour glucose (*β* = 0.46, *P* = 0.00090). However, only the latter remained significant after applying the Bonferroni correction. We note that most studies investigating association between *CRP* polymorphisms and fasting glucose did not reveal significant findings^29–32^. Furthermore, few studies examined association of *CRP* gene with 2-hour glucose. To our knowledge, this is the first report of a significant association between *CRP* polymorphism and 2-hour glucose. Since 2-hour glucose is an indicator of glucose tolerance, our finding suggests an association of *CRP* gene with glucose intolerance. In fact, we note that *CRP* gene is located within the chromosome 1q21-q25 and this region has been linked to type 2 diabetes and 2-hour glucose by several linkage studies^48^. Therefore, in addition to the modest associations between *CRP* polymorphisms and diabetes reported previously^24–27, 46, 47^, the significant association of rs1205 with 2-hour glucose observed in this study might increase the necessity of further efforts to investigate whether *CRP* gene plays a role in the pathogenesis of diabetes.

There are some limitations in this study. Firstly, this is a cross-sectional study and no causal inferences about the observed associations can be made. Further longitudinal studies are needed to dissect cause-and-effect mechanisms of these observed associations. Secondly, the information on glycated hemoglobin (HbA1c) was not collected in the study sample, which may better reflect abnormal glucose metabolism and hyperglycemia than either fasting or 2-hour glucose. Since HbA1c level is also a criterion to diabetes diagnosis^11^, we recognize that lack of this information might result in that some diabetes cases in the study sample were not identified, which could influence the results of association with diabetes. Besides, we do not have information about some confounding factors of CRP level, such as bacterial infection, autoimmune, and so forth. Since we successfully replicated the associations of CRP levels with *CRP* SNPs, diabetes, and glucose levels reported previously, the influence of lack of these confounding factors on our analysis might be not severe. Furthermore, the inconsistent and modest associations of *CRP* polymorphisms with diabetes and glucose levels observed in previous and current studies could be due to insufficient sample size. Since the sample recruitment of the SAPPHIRe study has been terminated, the sample size of our study is limited. Further studies with larger sample or collaboration of multiple groups are necessary to investigate these indefinite associations.

In conclusion, in addition to successfully replicating the well-known associations of *CRP* SNPs with CRP levels and associations of CRP levels with diabetes and glucose levels, this study revealed a novel significant association between a *CRP* polymorphism and 2-hour post challenge glucose in a Taiwan population. Since few studies have investigated the association between *CRP* gene and 2-hour glucose and ethnic heterogeneity may exist in genetic determinants of glucose levels, our finding has to be confirmed in other populations. Further efforts are needed not only to dissect the mechanism underlying the association between *CRP* gene and 2-hour glucose but also to investigate whether *CRP* gene plays a role in the pathogenesis of diabetes.

## Methods

### Study design and study subjects

This is a cross-sectional study. The study subjects came from the SAPPHIRe Taiwan follow-up study. Briefly, the SAPPHIRe study was conducted to identify genetic determinants that influence susceptibility to hypertension and insulin resistance based on concordant sibpairs (both sibs with hypertension) and discordant sibpairs (one hypertensive and one hypotensive sib). The SAPPHIRe network recruited subjects with hypertension and their family members of either Chinese or Japanese descent at six sites in Taiwan, Hawaii, and the California Bay area from 1995 to 2000^49^. In 2001, the SAPPHIRe Taiwan follow-up study was conducted^50^. This follow-up study consists of 1,093 SAPPHIRe Taiwanese participants and 945 of whom were siblings. The current study was based on the genotypes of *CRP* SNPs of these 945 siblings and their clinical data collected during the period of follow-up study, from 2001 to 2005.

This study was approved by the institutional review boards of the National Health Research Institutes, National Taiwan University Hospital, Tri-Service General Hospital, Taipei Veterans General Hospital, and Taichung Veterans General Hospital. All participants signed informed consent forms at study entry.

### Clinical measures and lifestyle factors

The participants received anthropometric measurements at 8 a.m. after 8–10 hours overnight fast and without wearing shoes or heavy clothes. Body mass index (BMI) was calculated as weight in kilograms divided by the square of height in meters (kg/m^2^). Furthermore, waist circumference was also measured. Overnight fasting blood samples were collected after the anthropometric measurements. The concentrations of CRP, glucose, total cholesterol (CHOL), HDL-cholesterol (HDL), and triglycerides (TG) were measured in fasting samples. Serum high-sensitivity CRP level was measured with a latex particle enhanced immunoturbidimetry (LIT) kit (Good Biotech Corp, Taichung, Taiwan) as described previously^23^. A CRP measure larger than or equal to 10 mg/L may indicate acute clinical inflammation and subjects whose CRP level larger than or equal to 10 mg/L were excluded from association analysis related to circulating CRP. A standard 75-g oral glucose tolerance test (OGTT) was performed, and the post-challenge plasma glucose was measured after two hours. Subjects meeting the following criteria were defined as having diabetes: (1) fasting glucose level ≥ 7 mmol/L, (2) 2-hour glucose level ≥ 11.1 mmol/L during OGTT, or (3) taking medications for treating diabetes. Systolic blood pressure (SBP) and diastolic blood pressure (DBP) was measured after subjects had been sitting at rest for 10 minutes. Subjects meeting the following criteria were defined as having hypertension: (1) having SBP ≥ 140 mmHg or DBP ≥ 90 mmHg, or (2) taking at least one medication for controlling high blood pressure. History of cardiovascular diseases including stroke and heart disease and lifestyle factors including cigarette smoking, alcohol drinking, and physical activity, were also obtained by questionnaire. Smoking status was categorized as either never-smoker or current- or ever-smoker. Alcohol-drinking status was categorized as either never-drinker or current- or ever-drinker. For physical activity, subjects were categorized as sedentary or non-sedentary^51^.

### SNP selection and genotyping

**Figure 1:**
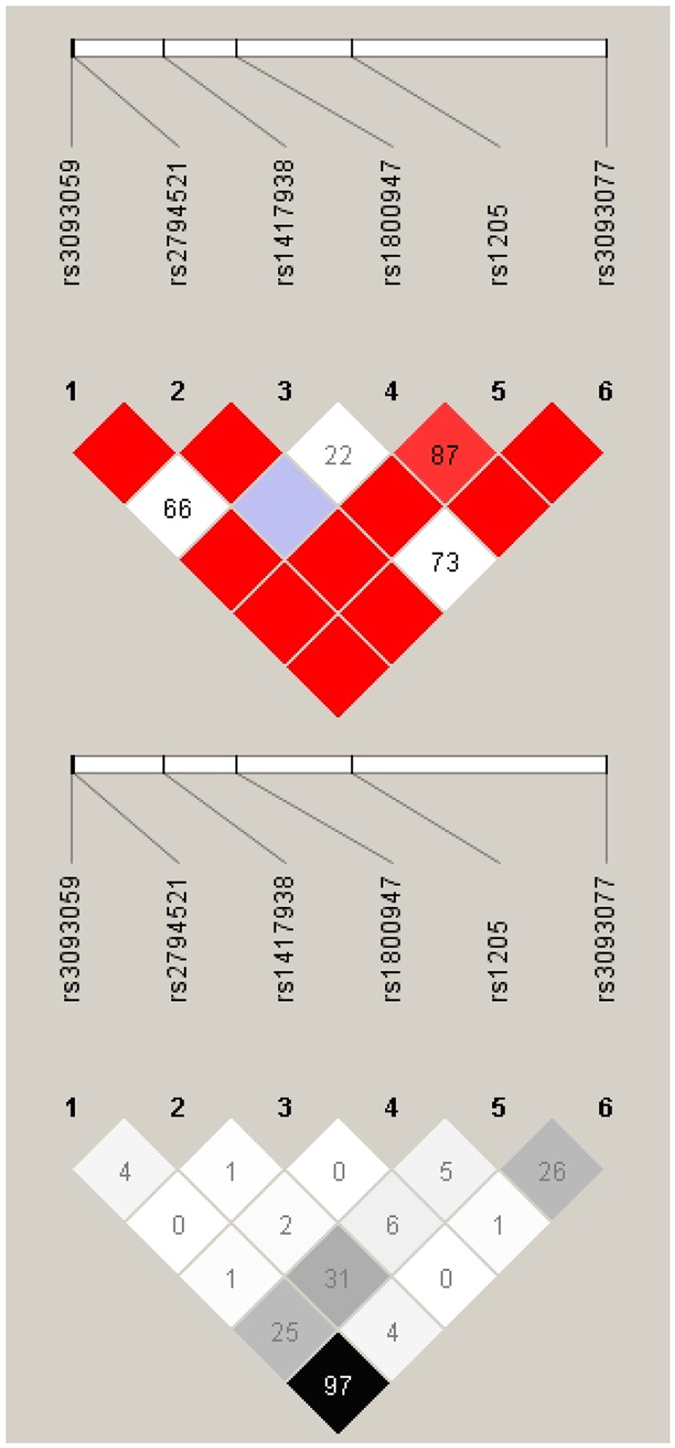
Linkage disequilibrium (LD) structure of the *CRP* tag-SNPs genotyped in the sample of SAPPHIRe study. The LD structure was analyzed using the Haploview (v. 4.2) program. The upper diagram presents the pair-wise LD levels measured by Lewontin’s |D’|, while the lower diagram presents the pair-wise LD levels measured by *r* ^2^.

In the SAPPHIRe study, genotyping of *CRP* SNPs was implemented in a set of 1,234 subjects, which contained the 945 siblings in the follow-up study. Initially, six SNPs of the *CRP* gene with minor allele frequency (MAF) > 5% or that had been reported previously in publications were selected for genotyping. These six SNPs are rs3093059 (T-C base change in the promoter region, AF449713 position 969), rs2794521 (A-G base change in the promoter region, AF449713 position 1009), rs1417938 (A-T base change in the intron region, AF449713 position 1919), rs1800947 (G-C base change at codon 184, AF449713 position 2667), rs1205 (G-A base change in the 3′ untranslated region (UTR), AF449713 position 3872), and rs3093077 (G-T base change in the 3′ UTR, AF449713 position 6469). According to the genotype data of CHB population from HapMap 3 release 28, these six SNPs capture more than 87.5% of common *CRP* SNPs (MAF > 0.01) at *r* ^2^ ≥ 0.8 in the Chinese population. Information on *CRP* genotype data obtained in the SAPPHIRe study is reported in Table 2. The linkage disequilibrium (LD) structure among these SNPs is represented in Fig. 1.

Genomic DNA was extracted from peripheral lymphocytes using a Puregene Kit (Minneapolis, Minn., USA). All SNPs were genotyped using a genotyping platform based on the 5′ nuclease allelic discrimination Taqman assay in a 96-well format on the ABI Prism 7000HT Sequence Detection System (Applied Biosystems). The PCR primers and probes for individual SNPs were designed using the Assays-by-Design Service (Applied Biosystems).

All the methods were performed according to the approved guidelines and regulations. All experimental protocols were approved by committee of National Health Research Institutes, National Taiwan University Hospital, Tri-Serve General Hospital, Taipei Veterans General Hospital, and Taichung Veterans General Hospital.

### Statistical analysis

The clinical characteristics of the study subjects were given as follows. Quantitative variables, except for levels of CRP and TG, were expressed as mean ± standard deviation (SD). Due to the positively skewed distribution, the CRP and TG levels were expressed as geometric mean with interquartile range (IQR). Qualitative variables were presented in percentages.

For the assayed SNPs, the estimation of MAF, the test for Hardy-Weinberg equilibrium (HWE), and the measure of LD between any pair of SNPs were performed by using Haploview software^52^. Furthermore, the pairwise tagging option of Haploview was implemented to select tag-SNPs.

Throughout our association analyses, the CRP level was logarithmically transformed. The associations of individual tag-SNPs with the level of circulating CRP were assessed by multiple linear regression models and were adjusted for gender, age, BMI, waist, SBP, DBP, hypertension, diabetes, CVD, CHOL, HDL, TG, physical activity, smoking, and alcohol drinking. The associations of CRP level and associated tag-SNPs with diabetes prevalence were investigated by multiple logistic regression models, which incorporated gender, age, BMI, waist, SBP, DBP, hypertension, CVD, CHOL, HDL, TG, physical activity, smoking, and alcohol drinking as the covariates for adjustment. On the other hand, multiple linear regression models were used to examine the associations of CRP level and associated tag-SNPs on the levels of fasting and 2-hour glucose, and the adjusted covariates included gender, age, BMI, waist, SBP, DBP, hypertension, CVD, CHOL, HDL, TG, physical activity, smoking, alcohol drinking, and the use of medications for treating diabetes. The associations of tag-SNPs on the clinical variables were investigated under the additive genetic model. To deal with the correlation between members within the same families, the GEE approach was used throughout the association analyses and was implemented by using SPSS Version 18.0. In each GEE analysis, two-sided test was conducted, the significance level of 0.05 was used, and the Bonferroni correction was applied to deal with multiple testing.

To demonstrate that significant SNP-trait associations observed in GEE analysis were not caused by population stratification, the observed associations were re-examined by the FBAT analysis^53^, which has the advantage of avoiding confounding due to population stratification. The software FBAT 2.0.4 was used to implement the FBAT analysis and one-sided p-value was calculated.

## Electronic supplementary material

Supplementary Table S1
