# Supplementary material for: CRP-level-associated polymorphism rs1205 within the CRP gene is associated with 2-hour glucose level: The SAPPHIRe study
Source: Sci Rep. 2017 Aug 11;7:7987. doi: 10.1038/s41598-017-08696-2 (PMC5554245; doi:10.1038/s41598-017-08696-2)
Supplement: Supplementary file 1 — Supplementary Table S1 [file 41598_2017_8696_MOESM1_ESM.pdf]

Supplementary Information

CRP-level–associated polymorphism rs1205 within the *CRP* gene is associated with 2-hour glucose level: The SAPPHIRe study

Wayne Huey-Herng Sheu[1,2,3,4], Wen-Chang Wang[5,6], Kwan-Dun Wu[7], Chih-Tsueng He[8], Chii-Min Hwu[9,10], Thomas Quertermous[11], Wan-Shan Hsieh[6], Wen-Jane Lee[12], Chih-Tai Ting[13], Yii-Der I. Chen[14], Chao A. Hsiung[6]

1 Division of Endocrinology and Metabolism, Department of Internal Medicine, Taichung Veterans General Hospital, Taichung, Taiwan.

2 College of Medicine, National Yang-Ming University, Taipei, Taiwan.

3 Institute of Medical Technology, National Chung-Hsing University, Taichung, Taiwan.

4 School of Medicine, National Defense Medical Center, Taipei, Taiwan.

5 The Ph.D. Program for Translational Medicine, College of Medical Science and Technology, Taipei Medical University, Taipei, Taiwan.

6 Division of Biostatistics and Bioinformatics, Institute of Population Health Sciences, National Health Research Institutes, Zhunan, Miaoli County, Taiwan.

7 Department of Internal Medicine, National Taiwan University Hospital, Taipei, Taiwan.

[8] Division of Endocrinology & Metabolism, Tri-Service General Hospital, Taipei, Taiwan.

[9] Section of Endocrinology and Metabolism, Department of Medicine, Taipei Veterans General Hospital, Taipei, Taiwan.

[10] Faculty of Medicine, School of Medicine, National Yang-Ming University, Taipei, Taiwan.

[11] Division of Cardiovascular Medicine, Falk Cardiovascular Research Building, Stanford University School of Medicine, Stanford, CA, USA.

[12] Department of Medical Research, Taichung Veterans General Hospital, Taichung, Taiwan.

[13] Cardiovascular Center, Taichung Veterans General Hospital, Taichung, Taiwan.

[14] Los Angeles Biomedical Research Institute, Harbor-UCLA Medical Center, Torrance, California, USA.

Supplementary Table S1. FBAT analysis for the association of minor allele *G* at rs1205 with levels of CRP and 2-hour glucose.

Trait	FBAT statistic, *Z*	P-value of one-sided test
Circulating CRP[a]	2.049	0.020
2-hour glucose[b]	2.726	0.0032

[a]Prior to FBAT analysis, the log-transformed CRP levels were adjusted for gender, age, BMI, waist, SBP, DBP, hypertension, diabetes, CVD, CHOL, HDL, TG, physical activity, smoking, and alcohol drinking. The residuals were used as the trait in the FBAT analysis.

[b]Prior to FBAT analysis, the 2-hour glucose levels were adjusted for gender, age, BMI, waist, SBP, DBP, hypertension, CVD, CHOL, HDL, TG, physical activity, smoking, alcohol drinking, and the use of medications for treating diabetes. The residuals were used as the trait in the FBAT analysis.
